# Quickest detection of drug-resistant seizures: An optimal control approach

**DOI:** 10.1016/j.yebeh.2011.08.041

**Published:** 2011-12

**Authors:** Sabato Santaniello, Samuel P. Burns, Alexandra J. Golby, Jedediah M. Singer, William S. Anderson, Sridevi V. Sarma

**Affiliations:** a Department of Biomedical Engineering, Johns Hopkins University, Baltimore, MD, USA; b Department of Neurosurgery and Department of Radiology, Brigham and Women's Hospital, Boston, MA, USA; c Department of Ophthalmology and Neurology, Children's Hospital, Boston, MA, USA; d Department of Neurosurgery, Johns Hopkins Hospital, Baltimore, MD, USA

**Keywords:** Quickest detection, Bayesian estimation, Multivariate analysis, Intracranial electroencephalogram, Optimal control, Hidden Markov model, Dynamic programming, Networks

## Abstract

Epilepsy affects 50 million people worldwide, and seizures in 30% of the cases remain drug resistant. This has increased interest in responsive neurostimulation, which is most effective when administered during seizure onset. We propose a novel framework for seizure onset detection that involves (i) constructing statistics from multichannel intracranial EEG (iEEG) to distinguish nonictal versus ictal states; (ii) modeling the dynamics of these statistics in each state and the state transitions; you can remove this word if there is no room. (iii) developing an optimal control-based “quickest detection” (QD) strategy to estimate the transition times from nonictal to ictal states from sequential iEEG measurements. The QD strategy minimizes a cost function of detection delay and false positive probability. The solution is a threshold that non-monotonically decreases over time and avoids responding to rare events that normally trigger false positives. We applied QD to four drug resistant epileptic patients (168 hour continuous recordings, 26–44 electrodes, 33 seizures) and achieved 100% sensitivity with low false positive rates (0.16 false positive/hour). This article is part of a Supplemental Special Issue entitled The Future of Automated Seizure Detection and Prediction.

## Highlights

► A control-theoretical framework for automatic online seizure detection is proposed. ► This framework combines iEEGs, network-based statistics, and optimization tools. ► The detection algorithm minimizes detection delays and probability of false alarms. ► Reported results show 100% sensitivity and low false positive rates.

## 1 Introduction

Automatic online seizure detection (AOSD) in intractable epilepsy has generated great interest in the last 20 years and is a fundamental step toward the development of neurostimulation-based responsive antiepileptic therapies [1–3]. Pioneering works in the late 1970s and early 1980s by Gotman et al. [4,5] showed that seizures can be automatically separated from interictal activity, and since then, several approaches to AOSD have been proposed by exploiting either scalp or intracranial EEG recordings, single or multichannel analysis, linear or nonlinear features.

Osorio et al. [6–9] used a wavelet-based decomposition of selected intracranial EEG recordings (iEEGs) to (i) separate the seizure-related component from the background noise, (ii) track the ratio between these components in the time–frequency domain, and (iii) detect a seizure when such a ratio crosses a fixed threshold for a sufficiently long time. Parameters of the detection method (e.g., threshold, duration of the suprathreshold condition, etc.) can be either fixed [6] or adaptive [7,8]. Fixed threshold-based approaches were also proposed in [10–12], where the threshold was applied to linear spectral features of the iEEGs.

Gotman et al. [13–15] proposed a probabilistic framework for seizure detection using both scalp EEGs [15] and iEEGs [13,14]. In this framework, amplitude and energy measures in multiple frequency bands are computed for each channel via wavelet decomposition and the corresponding sampled probability distribution function is estimated. Then, the probability of a seizure is conditioned to the value of such measures and estimated via Bayes’ rule. A patient-specific threshold is finally applied on this conditional probability of seizure to decide, for each channel, whether a seizure is likely or not, and a seizure is detected when that threshold is passed in a sufficient number of channels.

In all the studies mentioned above, the AOSD was solved by (i) introducing a relevant statistic that is computed from the available measurements and that captures changes in brain activity at the seizure onset, and (ii) a rule that, based on this statistic, determines whether a seizure has occurred or not.

More recently, AOSD has been implemented using sophisticated classification tools. In particular, EEG channels (either scalp or intracortical) have been processed individually to extract multiple univariate or bivariate features in time, frequency, or wavelet domain [16–32]. Then, for each channel, the available features have been combined into vectors and classified via support vector machines (SVM) [21,23,25,29], principal component analysis (PCA) [27,28], artificial neural networks (ANN) [16,18–20,24,30–32], fuzzy logics [22], or pattern recognition tools [17]. Finally, decisions made for different channels have been combined or ranked to ultimately determine whether a seizure has occurred or not. As a variation to this paradigm, [26,33] merged features extracted from different channels into one vector and applied the classification rule to this vector.

Although many of the aforementioned methods have sensitivity well above 90%, results generally show lower specificity (i.e., higher number of false positives) when applied to test data, and a comparative study by Lee et al. [34] reported performance varying from patient to patient. This may not be surprising considering that (i) several parameters in these methods are patient specific and tuned heuristically; and (ii) these methods do not explicitly minimize relevant detection performance criteria (e.g., delay or probability of false positives). The latter drawback reflects the important fact that current detection paradigms develop algorithms first which then define and limit the performances. We believe that performance specifications should be stated up front in a cost function to be minimized (e.g., delay length, false positive rate, etc.) which then defines the algorithm.

We propose a novel computational framework for AOSD that involves (a) constructing network-based statistics from multichannel neural data to distinguish nonictal from ictal states; (b) modeling the evolution of such statistics in nonictal and ictal states and their transition probabilities; and (c) developing an optimal model-based strategy that detects the transitions from nonictal to ictal states by using sequential multichannel measurements. The combination of (a) and (b) results in a *dynamic detector,* which, unlike a standard classifier, evolves over time based on current and past measurements, thus automatically adapting to brain dynamics.

In our formulation, AOSD is posed as a change-point detection problem and solved by minimizing a cost function of the average distance between actual and estimated seizure onset, the probability of false positives and the probability of delayed detection. This formulation is a variation of the “Quickest Detection” (QD) [35,36], whose theory we extended to allow for different cost functions and, more importantly, for dependent measurements, which is more applicable to neural data [37].

We applied our framework to four subjects with drug-resistant seizures (168 hours [h] of continuous recordings from 26–44 iEEG electrodes, 33 seizures), which resulted in 100% sensitivity on both training and validation data, low false-positive rate (min: 0.16 false positive/hour [FP/h]; max: 2.95 FP/h; mean: 1.39 FP/h), and an average detection delay of 9.6 seconds. Compared with a standard Bayesian and a heuristic threshold detector (i.e., non-optimal policies for which no explicit cost function is minimized), our approach showed lower false-positive rates and higher sensitivity.

## 2 Methods

### 2.1 Clinical data

**Fig. 1:**
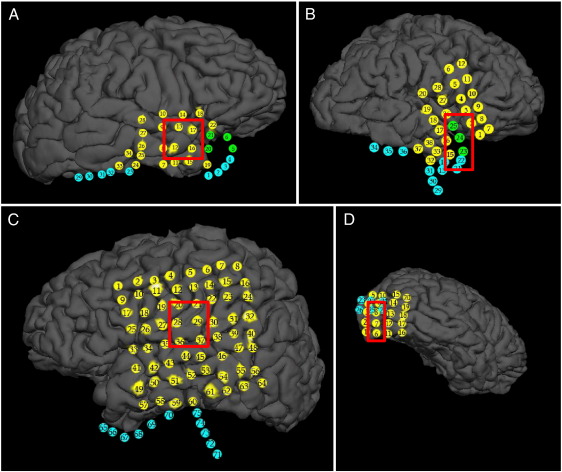
Three-dimensional reconstructions of the brain for patients 1 (A), 2 (B), 3 (C), and 4 (D) with the electrode grids superimposed. Red-squared boxes denote the hand-annotated seizure foci. In (A) to (D), yellow circles are electrodes visible in their proper location on the cortex; green circles are electrodes that would be visible where the underlying cortex rendered properly; cyan circles are electrodes occluded by cortex (rendered or not) because they wrap around underneath or behind the temporal lobe. In patient 2, recordings from electrodes 1, 4, 6, 23, and 33–37 were not included in this study. In patient 3, recordings from electrode 34–64 were not included in this study. Electrodes not included in this study showed neglegible modulation during the ictal periods.

Four subjects with intractable epilepsy were surgically implanted with subdural grid and strip electrodes (26–75 channels, Ad-Tech® Medical Instrument Corp., Racine, WI, USA) for approximately 1 week before surgical resection of the focal region and monitored by clinicians for seizures and interictal epileptic activity. Electrodes are 4 mm diameter platinum contacts embedded in a silicone sheet with 2.3 mm exposed. Data were digitized and stored using an XLTEK® EMU128FS system (Natus Medical Incorporated, San Carlos, CA, USA) with 250 to 500 Hz sampling frequency. Table 1 and Fig. 1 report patient-specific information, number of electrodes included in this study, and electrode position.

Board-certified electroencephalographers (up to three) marked, by consensus, the unequivocal electrographic onset (UEO) [38] of each seizure and the period between seizure onset and termination. The time of seizure onset was indicated by a variety of stereotypical electrographic features, which could include, but were not limited to, the onset of fast rhythmic activity, an isolated spike or spike–wave complex followed by rhythmic activity, or an electrodecremental response. These features were typically present in one to a few channels at ictal onset. At all times, concurrent changes in the patient's behavior were sought from the video segment of a video-EEG recording. UEOs were used as the “gold standard” for evaluating the performance of the detection algorithm. Electrode recordings (iEEG in the following) included in this study were made available to the authors with the written consent of the patients, in accordance with the protocol approved by the institutional review boards at Brigham and Women's Hospital and Children's Hospital, Boston, MA, USA. Recordings included in this study cover a period of two consecutive days per patient (days with seizures).

### 2.2 Multichannel analysis

Recent studies have introduced schemes that analyze all the available electrode channels simultaneously [39–48]. In these schemes, each electrode is treated as a node in a graph, and any two nodes are considered *connected* (i.e., an edge exists between them) if the activities at these sites are dependent. The connectivity (topology) of the graph can then be described by a matrix. Statistics computed from this matrix (which is referred to as “connectivity” or “adjacency” matrix [49]) can show if the topology changes significantly from nonictal to ictal states or vice versa, and significant changes in these statistics can be used to detect a seizure onset.

In order to capture potential linear dependencies between all of the recording sites, we computed the connectivity matrix, *A*, as the cross-power in a chosen frequency band (theta, alpha, etc.) between the available iEEG channels. That is, for each pair of channels (*i*, *j*) the corresponding element of the connectivity matrix, *A*_*ij*_, was

(1) $$A_{ij}≜\int_{lb}^{ub}P_{ij}\left(\omega \right)d\omega \;$$

where *P*_*ij*_(*ω*) is the cross-power spectral density of channels *i* and *j* at frequency *ω* [50]. The frequency band [*lb*, *ub*] in (1) was patient specific and chosen among theta (4–7 Hz), alpha (8–13 Hz), and beta (14–30 Hz) bands (see Table 1 for the selection criteria) in our data set. Finally, the matrix *A* was computed over a 5-second-long sliding window (1-second [s] slide), resulting in a sequence of matrices {*A*(*k*)}, one for each time second *k*.

#### 2.2.1 Singular value decomposition

It has been reported that the brain enters a more organized, lower-complexity state prior to a seizure [51,52]. As the brain becomes more organized and nodes become more connected, the *rank* (number of linearly independent rows or columns) of the connectivity matrix drops. The singular value decomposition (SVD) of a matrix highlights the rank of a matrix and also generates a weighted set of vectors that span the range space and null space of the matrix [53]. We therefore used SVD to measure the time-varying complexity of the brain by tracking the rank and its associated subspaces as a means to characterize nonictal versus ictal states.

The SVD of a generic *m* × *n* connectivity matrix *A* is defined as (2)where *U* is an *m* × *m* unitary (UU* = *I*) matrix whose columns, ***u***_*i*_, are the eigenvectors of the matrix AA*, *V* is an *n* × *n* unitary matrix whose columns, ***v***_*i*_, are the eigenvectors of the matrix A*A, and * denotes the complex conjugate transpose operator. *S* is an *m* × *n* matrix whose first *r* diagonal entries *σ*_1_ ≥ *σ*_2_ ≥ … ≥ *σ*_*r*_ are the nonzero singular values of *A*, with *r* being the rank of *A* [53]. The first *r* columns of *U* span the column space of *A* and the first *r* rows of *V* span the row space of *A*. When *m* = *n* and *A* is symmetric, the SVD reduces to the conventional eigenvalue decomposition, where the singular values are the square of the eigenvalues of *A, U = V*^− 1^, and the columns of *U* and *V* are the eigenvectors of *A* [53].

**Fig. 2:**
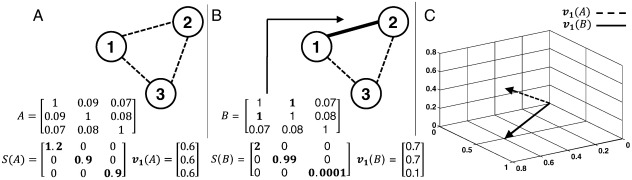
(A) Three nodes in a network are loosely connected and the correspondent connectivity matrix *A* is close to identity with full rank. (B) Two of three nodes are strongly connected and the connectivity matrix *B* is close to losing rank. (C) Average direction and amplification of the first singular vectors corresponding to matrices *A* and *B*.

An example is shown in Fig. 2. Here, two three-node graphs are analyzed. In Fig. 2A all the nodes have similar weak connections (strength ≤ 1). The SVD of the corresponding connectivity matrix, *A*, reveals that the matrix of this graph has full rank (three non-zeros and comparable singular values). More physically, full rank here indicates that the activity in the three nodes spans a three-dimensional space, or has 3 degrees of freedom.

If one of the connections is strengthened, as is the case between nodes 1 and 2 (Fig. 2B)*,* one of the singular values of the corresponding connectivity matrix, *B*, becomes small in comparison to the other two, indicating that the rank of matrix has approximately dropped to 2. This means that with the addition of one strong connection, the activity of the graph collapses to two dimensions and become more “ordered”. The singular vectors of graphs in Figs. 2A and B are plotted in Fig. 2C and indicate that the dominant direction of the vectors has rotated and the average amplification of the connectivity matrix has increased when the strong connection is added. We investigate the first (i.e., maximum) singular value, *σ*_1_(*k*), from each connectivity matrix *A*(*k*) computed in (1) and apply QD on this statistic.

### 2.3 Quickest detection of seizure onsets

#### 2.3.1 Hidden Markov model estimation

**Fig. 3:**
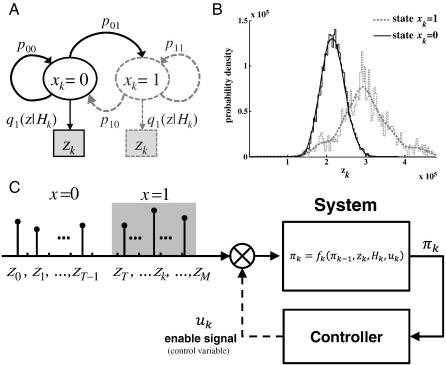
(A) Schematic of an *m* = 2 state HMM. (B) Sampling probability distribution function of the observations *z*_*k*_ in state *x*_*k*_ = 0 (nonictal) and *x*_*k*_ = 1 (ictal). Data collected from patient 2. (C) Seizure onset detection formulated as a feedback control problem.

For any given patient, we assume that the maximum singular value computed at each second (observations), *z*_*k*_ ≜ *σ*_1_(*k*), *k* = 1, 2, …, is generated by a hidden Markov model (HMM) [54]. At each stage *k* ≥ 0, the brain is in one of *m* states, that is, *x*_*k*_ ∈ {0, 1, …, *m*− 1}, which follows a Markov chain [54],

$$\mathrm{Pr}\left(x_{k+1}=j\text{|}x_{k}=i,x_{k-1},\ldots ,x_{0}\right)=\mathrm{Pr}\left(x_{k+1}=j\text{|}x_{k}=i\right)≜p_{\mathrm{ij}},$$

(3) $$\sum_{h=1}^{m-1}p_{\mathrm{ih}}=1\;p_{0}+p_{1}+\cdots +p_{m-1}=1\text{,}$$

for all *i*,*j* = 0,1,2,..., *m*−1, where $\;p_{i}≜\mathrm{Pr}\left(x_{0}=i\right)\text{,}\;i=0,1,\ldots ,m-1$, is the probability of starting in state *i*. For a fixed state *i*, the observations *z*_*k*_ are generated according to a known history-dependent probability law *q*_*i*_(*z*|*H*_*k*_) ≜ Pr(*z*_*k*_ = *z*|*x*_*k*_ = *i*, *H*_*k*_), where *H*_*k*_ ≜ {*z*_0_, *z*_1_, …, *z*_*k*− 1_} denotes the sequence of past observations. Note that the dependency of *z*_*k*_ on previous observations is introduced to account for temporal dependencies that exist in neural data. The HMM is uniquely defined by the triple {P,Σ,q}, with$\;P≜\left[p_{0}p_{1}\ldots p_{m-1}\right]\text{,}$*Σ*_*ij*_ ≜ *p*_*ij*_, *i*, *j* = 0, 1, …, *m*− 1, and *q* ≜ [*q*_0_ … *q*_*m*− 1_]. See Fig. 3A for a schematic of a *m* = 2 state HMM. For our QD framework, we fitted a *m* = 2 state HMM on each patient, with state *x* = 0 and *x* = 1 denoting the nonictal and ictal conditions, respectively (Fig. 3B). The ictal state begins and ends with the unequivocal ictal onset and offset determined by clinicians. Early ictal or preictal conditions were subsumed in the nonictal state as they may not exist in all patients.

Because we begin monitoring a patient in the nonictal state 0, we set $\;P=\left[1\;0\right]$. We also assume that the state transition probability matrix is

(4) $$\text{Σ}=\left[\begin{array}{cc} 1-\rho & \rho \\ 0 & 1 \end{array}\right]\;$$

where *ρ* was estimated from training data via maximum likelihood estimation [55]. The output probability law *q*_*x*_(*z*|*H*_*k*_), *x* = 0, 1 was computed by combining generalized linear models (GLMs) [56] and maximum likelihood estimation. Observations were first quantized and mapped to integer nonnegative numbers in order to have a discrete observation domain, i.e., $\;Q\left(\left[z_{k}\right]\right)≜n_{k}$ with $\;n_{k}\in Z_{0}^{+}$, for all *k*. Then, we assumed that the probability mass function of the sequence *n*_*k*_ follows the Poisson law,

(5) $$q_{x}\left(z\text{|}H_{k}\right)≅\text{Pr}\left(n_{k}=Q\left(\left[z\right]\right)\text{|}H_{k},x\right)≜e^{-\lambda_{x,k}}\frac{\lambda_{x,k}{}^{Q\left(\left[z\right]\right)}}{Q\left(\left[z\right]\right)!}\;$$

where the instantaneous rate *λ*_*x*,*k*_ depends on the current state *x*, stage *k*, and history *H*_*k*_. Finally, we assumed *λ*_*x*,*k*_ is given by the GLM

(6) $$\log\lambda_{x,k}=\alpha_{x}+\sum_{j=1}^{L}\beta_{x,j}n_{k-j}$$

where Θ_*x*_ ≜ {*α*_*x*_, *β*_*x*, 1_, …, *β*_*x*, *L*_} is a vector of parameters to be fitted on the data via maximum likelihood estimation. The number *L* = 15 of past observations to be used in (6) determines the number of parameters in Θ_*x*_ and was chosen by minimizing the Akaike's information criterion (AIC) [57] over a set of candidate models. For each patient, the parameter vector Θ_*x*_ was estimated separately for state *x* = 0 and *x* = 1 on training data. Such data included 3 h of continuous interictal recordings well before seizure (min 3 h, max 12 h before the seizure) and 1 (patients 1–3) or 2 (patient 4) hand-annotated ictal periods.

#### 2.3.2 HMM state evolution and QD policy

Because the state of a HMM is hidden, we introduce the Bayesian *information state variable π*_*k*_ ≜ Pr(*x*_*k*_ = 1|*z*_*k*_,*H*_*k*_) [37,58] in order to estimate how likely the transition from the nonictal to ictal state is at each stage *k*. Note that *π*_*k*_ is the *a posteriori* probability of being in state 1 at stage *k* and depends on the observations up to and including stage *k*. The evolution equation of *π*_*k*_ is recursive and given by

(7) $$\pi_{k+1}=\frac{L_{k+1}\left[\pi_{k}+(1-\pi_{k})p_{01}\right]}{\left(1-\pi_{k}\right)(1-p_{01})+L_{k+1}\left[\pi_{k}+(1-\pi_{k})p_{01}\right]}≜{\text{Φ}}_{k}\left(\pi_{k},z_{k+1},H_{k+1}\right)\;$$

with initial condition

$$\pi_{0}=P\left(x_{0}=1\text{|}z_{0}\right)=\frac{q_{1}\left(z_{0}\right)(1-p_{0})}{q_{0}\left(z_{0}\right)p_{0}+q_{1}\left(z_{0}\right)(1-p_{0})}$$

where *q*_*x*_(*z*_0_) is the probability of observing *z*_0_ in state ***x*** at time *k* = 0, *p*_01_ = *ρ* because of (4), and

$$L_{k}≜\frac{q_{1}(z_{k}|H_{k})}{q_{0}\left(z_{k},|,H_{k}\right)}$$

is the likelihood ratio. See Appendix for details.

The Quickest Detection (QD) problem is an *online* decision problem, where at each stage *k* we test the hypothesis H≜{aseizureonsethasoccurred} conditioned on the observations (*H*_*k*_, *z*_*k*_). We introduce the decision variable *u*_*k*_ ∈ {0, 1}, where *u*_*k*_ = 0 (*u*_*k*_ = 1) denotes that the hypothesis H is rejected (accepted) at stage *k*. In this way,

(8) $$\pi_{k+1}=f_{k}\left(\pi_{k},z_{k+1},H_{k+1},u_{k}\right)≜\{\begin{array}{cc} {\text{Φ}}_{k}\left(\pi_{k},z_{k+1},H_{k+1}\right) & u_{k}=0\\ terminate\&restart & u_{k}=1 \end{array}$$

where the “*terminate & restart*” state implies that we restart the detection algorithm after a seizure has been detected. With this setup, the detection problem boils down to *deciding when* to switch from *u*_*k*_ = 0 to *u*_*k*_ = 1 and claiming that a seizure has occurred.

We designed a decision strategy that minimizes the following cost function, which weighs the average detection delay and the probability of a false positive:

(9) $$J_{0}≜(1-\gamma )E_{T_{|T_{\mathrm{QD}}<T}}\left\{T-T_{\mathrm{QD}}\right\}+\gamma \;E_{T_{|T_{\mathrm{QD}}>T}}\left\{{\left(T_{\mathrm{QD}}-T\right)}^{2}\right\}$$

where *T* and *T*_*QD*_ denote the actual and estimated seizure onsets, respectively. *T* is a random variable whose distribution is defined by the HMM transition probabilities, i.e., *P*(*T* = *k*) = (1−*ρ*)^*k*− 1^*ρ*. It is important to note that a time-varying HMM would determine that $P\left(T=k\right)=\prod_{i=1}^{k-1}\left(1-p_{01}\left(i\right)\right)p_{01}(k)$.

*E*_*T*_|*T*_*QD*_ < *T*__{∙} and *E*_*T*_|*T*_*QD*_ > *T*__{∙} denote the expected value of the distance between *T*_*QD*_ and *T* in case of false positive (*T*_*QD*_ < *T*) and delayed detection (*T*_*QD*_ > *T*), respectively. Finally, the parameter *γ* ∈ [0,1] allows one to trade off false-positive and delayed detection, while the expected value *E*_*T*_{∙} accounts for the average temporal distance between actual and estimated seizure onset. Note that the distance *T*_*QD*_ − *T* is squared in (9) for *T*_*QD*_ > *T* as we intend to penalize more situations where *T*_*QD*_ >> *T*, which account for very late detections.

To construct our QD policy, we constructed the cost (9) as a function of the information state *π*_*k*_. To do so, we first defined a cost-per-stage, *G*_*k*_(*π*_*k*_, *u*_*k*_), that penalizes both rejecting H after the state transition has occurred (i.e., delay, *k* ≥ *T*) and accepting H before the actual state transition has occurred (i.e., false positive, *k* < *T*), and is 0 otherwise:

$$G_{k}(\pi_{k}\text{,}\;u_{k})≜\{\begin{array}{c} \gamma E_{T|k>T}k-T\}∙\pi_{k}u_{k}=0\\ \left(1-\gamma )E_{T|k<T}T-k\}∙(1-\pi_{k}\right)u_{k}=1\\ 0otherwise \end{array}$$

Then, the decision deals with choosing the stage *T*_*QD*_ > 0 such that the policy (*u*_1_ = 0, *u*_2_ = 0, …, *u*_*T*_*QD*_− 1_ = 0, *u*_*T*_*QD*__ = 1) minimizes the overall cost

(10) $$J_{0}≜E_{z_{0},\ldots ,z_{M}}\left[G_{M}\left(\pi_{M}\right)+\sum_{i=0}^{M-1}G_{i}\left(\pi_{i},u_{i}\right)\right]\;$$

where *G*_*M*_(*π*_*M*_) is the final stage cost for rejecting hypothesis H. *M* is the horizon over which a seizure must be detected, and we set *M* for each patient to be the value of the average inter-seizure interval. It is fairly straightforward to show that (10) is equivalent to (9) [59]. One can interpret the minimization of (10) with respect to the variable *u*_*k*_ given the evolution model (8), as an optimal feedback control problem where *u*_*k*_ is the control variable (Fig. 3C). As a control problem, this formulation can be solved recursively via dynamic programming [59], and leads to the optimal QD policy

$$T_{\mathrm{QD}}=min\left\{0<k<M|\pi_{k}>F_{k}(\pi_{k},z_{k},H_{k})\right\}$$

where *F*_*k*_(*π*_*k*_,*z*_*k*_,*H*_*k*_) is an adaptive threshold that depends on the current observation, history, and information state variable. There is no simple closed form expression for *F*_*k*_(∙) which is computed recursively and decreases over time non-monotonically as discussed below. Details on the derivation of *F*_*k*_(∙) can be obtained in [37].

#### 2.3.3 Significance and performance tests

For each patient, we compared the QD policy with a classic Bayesian estimator (BE) [58], which is widely used in the field of change-point detection [35,58]. We also compared the QD policy versus a threshold-based detector (HT), where the threshold is chosen heuristically. The formula for the estimated seizure onset with each of these predictors is

$$BE:T_{\mathrm{BE}}≜min\left\{k>0|\pi_{k}>0.5\right\}$$

$$HT:T_{\mathrm{HT}}≜min\left\{k>0|z_{k}>\bar{h}\right\}$$

where the threshold $\;\bar{h}$ is fixed. In particular, we chose $\bar{h}≜\mu_{z}+3\sigma_{z}$, where *μ*_*z*_ and *σ*_*z*_ are the mean and standard deviation (SD) of *z*_*k*_ over the 3-h nonictal training data, respectively.

For each detection policy, we measured the delay between each estimated seizure onset time and the correspondent UEO, the number of true positives (TP), false positives (FP), and false negatives (FN). Every detection was classified as TP or FP if an UEO occurred within 20 s from the detection time or not. This time was chosen to be comparable to [14]. Each UEO that was not detected was classified as FN. Results are summarized in Tables 2 and Table 3.

## 3 Results

### 3.1 Multichannel analysis

**Fig. 4:**
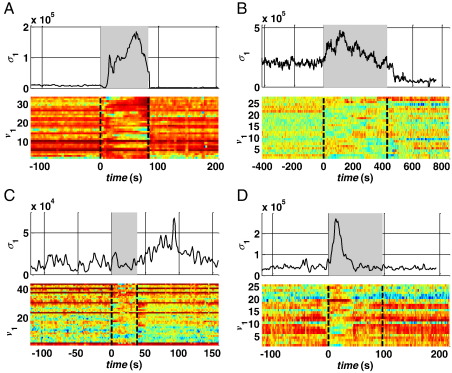
Sequence of first singular values *σ*_1_ and correspondent first singular vectors *v*_1_ around an ictal period (gray background) in patient 1 (seizure s_1_, A), 2 (s_1_, B), 3 (s_3_, C), and 4 (s_3_, D).

The maximum singular value *σ*_1_ estimated at each stage *k* from the connectivity matrix (1) is plotted in Fig. 4 (one ictal period per patient). The sequence of *σ*_1_ had a consistent pattern across the patients, with large values in nonictal state (pre- and post-seizure). The corresponding singular vector ***v***_1_ shows a leading direction before the seizure onset, which depends on both the specific patient and the location of the foci. For example, components 8–10 in patient 1 (Fig. 4A) and 1–4 in patient 3 (Fig. 4C) correspond to the focal area (Figs. 1A, C) and have significantly higher values than the other components of ***v***_1_ before the seizure.

During a seizure, however, *σ*_1_ rapidly increases compared with the nonictal background activity in the previous minutes, reaches a local maximum approximately half of the ictal period (gray boxes, Figs. 4A–D), and then slowly decreases to smaller, nonictal values. The change in the dynamics of *σ*_1_ was observed almost at the beginning of the hand-annotated seizure onset while the return to the nonictal condition was usually slower and may last longer than the hand-annotated end time of the seizure. Interestingly, after every seizure, *σ*_1_ decreased below the average value achieved before the seizure and, then, increased to the preictal values with a long drift (at least 2 hours, data not reported), which may be consistent with the definition of a postictal state given in [51,52]. The stereotypical dynamics of *σ*_1_ were associated with a sudden change in the direction of the singular vector ***v***_1_: the components with the largest values during the nonictal period decreased during the seizure while the remaining components increased. As a consequence, the distance between the largest and smallest components consistently decreased during a seizure (more than 30%), indicating a rotation of ***v***_1_ toward a new direction which varies with the specific seizure.

Modulo a scaling factor, the dynamics of σ1 and ***v***_1_ were similar in patients 1, 2, and 4 (Figs. 4A, B, D), independently of the type/origin of the seizure and the connectivity matrix (*σ*_1_ and ***v***_1_ were computed from connectivity matrices of cross-power in different frequency bands, see Table 1). These dynamics were less clear in patient 3, where *σ*_1_ showed slow oscillations independently of the seizure occurrence (Fig. 4C, top). However, at the seizure onset, the value of *σ*_1_ first decreased, then rapidly rose to a local maximum, and finally drifted toward the baseline value, as did occur in the other patients. This indicates that, modulo a scaling factor, the same dynamics occurred in patient 3 as well as patients 1, 2, and 4. Interestingly, in Fig. 4C (top), *σ*_1_ achieves a peak value ~ 50 s after termination of the seizure. However, this peak is shorter than the pattern during the previous seizure, is not followed by a recovery phase, and is not associated with a rotation of ***v***_1_. The leading components of ***v***_1_, indeed, do not change when this second peak occurs and are the same as in the preictal phase (Fig. 4C, bottom). These facts indicate that, although the absolute value of *σ*_1_ may be higher after the seizure, the specific pattern is different from the ictal one.

Overall, the clear modulation of *σ*_1_ at the seizure onset and its specific pattern during the ictal state may capture the overall change in complexity of the brain. This is possible because *σ*_1_ exploits information recorded from multiple sites simultaneously and combines linear dependencies between all the possible pairs of electrodes in the beta (patients 1, 3, 4) and theta (patient 2) frequency bands.

### 3.2 HMM estimation

**Fig. 5:**
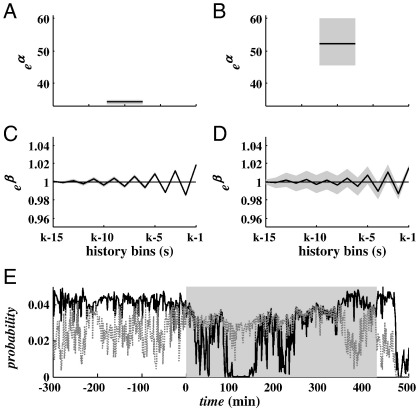
(A–D) Maximum likelihood estimate of parameters *Θ*_*x*_ (black line) in (6) with 95% confidence bounds (gray background) for nonictal (A, C) and ictal (B, D) state. (E) History-dependent estimate of the probability *q*_0_(*z*_*k*_|*H*_*k*_) (black line) and *q*_1_(*z*_*k*_|*H*_*k*_) (gray dotted line) around a specific hand-annotated seizure (gray background). Plots refer to patient 2. Probabilities in (E) refer to seizure *s*_3_ (validation data).

Figs. 3B and 5 show results for the HMM estimation. Although the mean value and the variance of *σ*_1_ were different in the ictal versus nonictal states, the sampling probability distribution functions had some overlap (Fig. 3B), which means that several of the same values of *σ*_1_ were likely to be achieved in both ictal and nonictal states.

In order to better characterize the distribution of *σ*_1_ in each state, we introduced a history-dependent representation of the probability distribution of *σ*_1_ (Fig. 5). At each stage *k*, the model (5)–(6) modulates the probability that the observation *σ*_1_(*k*) has been emitted while in the ictal or nonictal state based on the values of *σ*_1_ in the last 15 s. The dynamics of the instantaneous rate *λ*_*x,k*_ are captured by parameters Θ_*x*_ in (5) and (6), as Θ_*x*_ were estimated from actual observations in both states. Parameters in Figs. 5A–D indicate that the transition from nonictal to ictal state is characterized by (i) a significant increase in parameter *α*_*x*_ (Figs. 5A, B), which is a scaling factor and accounts for the average value of *σ*_1_ in state *x*, and (ii) larger 95% confidence bounds for parameters *β*_*x*, *j*_ (Figs. 5C, D), which accounts for larger differences between consecutive observations of *σ*_1_.

The different model parameters Θ_*x*_ resulted in distinct functions *q*_0_, *q*_1_, which (i) varied the probability of any given observation *σ*_1_ at each stage *k* depending on the past observations, and (ii) had opposite dynamics in the ictal and nonictal states (Fig. 5E). In particular, for the computed sequence of *σ*_1_ in each patient, *q*_1_(∙) was consistently larger than *q*_0_(∙) during the ictal periods, but decreased during the nonictal periods and was almost zero during the postictal phase, while *q*_0_(∙) was generally high (and almost always larger than *q*_1_(∙)) during the nonictal periods. In each patient, *q*_0_ and *q*_1_ were almost zero after every seizure independently of the model parameters, thus suggesting that the postictal period is characterized by a resetting of the brain activity, as argued in [51,52].

### 3.3 Quickest detection policy

**Fig. 6:**
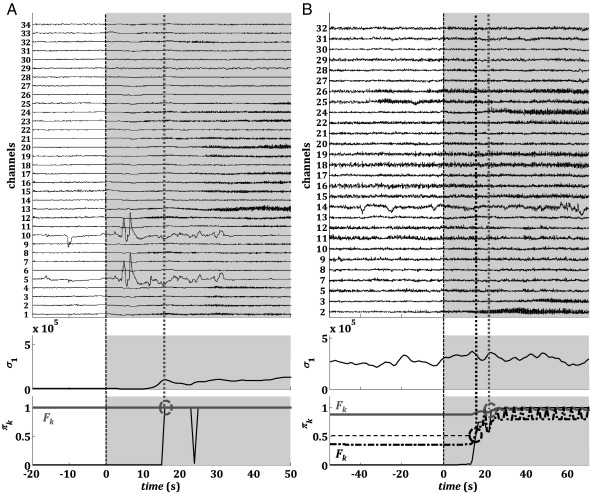
QD on validation data. The electrographic onset (dashed vertical black line), the correspondent QD estimation (circles), and threshold *F*_*k*_ (gray and dash-dot black lines) for patients 1 (A) and 2 (B). For patient 2, two QD estimations are computed for different values of parameter γ (black circle γ = 0.52; gray circle γ = 0.01). Plots refer to seizures *s*_1_ (patient 1) and *s*_3_ (patient 2), respectively. In (B) (bottom plot) the horizontal dashed black line denotes the threshold for the BE detector.

**Fig. 7:**
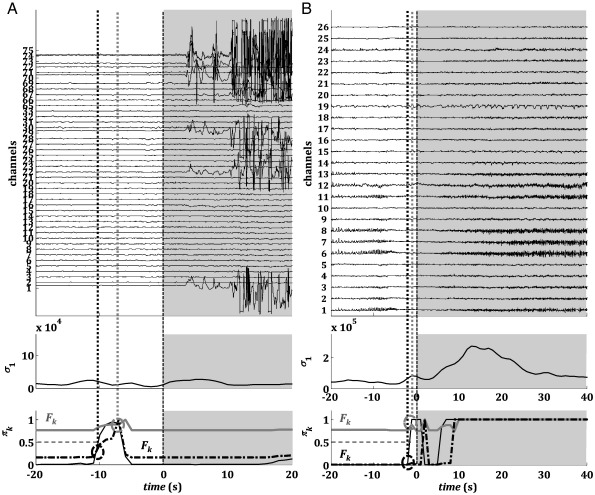
QD on validation data. The electrographic onset (dashed vertical black line), the correspondent QD estimation (circles), and threshold *F*_*k*_ (gray and dash-dot black lines) for patients 3 (A) and 4 (B). For each patient, two QD estimations are computed (gray and dash-dot black) for different values of parameter *γ*. Plots refer to seizures *s*_3_ (patient 3) and *s*_3_ (patient 4), respectively. In (A) and (B) (bottom plot) the horizontal dashed black line denotes the threshold for the BE detector.

Tables 2 and 3 and Figs. 6 and 7 report the results for the QD policy versus the Bayesian estimator (BE) and the heuristic threshold-based detector (HT) described under Methods.

The QD policy was derived by penalizing the probability of false positives more than the delay (i.e., γ < 0.5 in the cost function (9)). In this way, QD guaranteed 100% sensitivity (i.e., all the seizures were correctly detected) on both training and validation data (Table 2) and delays were generally small (average across patients: 9.6 ± 10.5 s, mean ± SD). Detection occurred earlier than the UEO (anticipation) in 5 of 28 seizures on validation data (Table 3), with a lag ranging from 4 to 7 s. The number of false positives, instead, varied with the patient (Table 2) and determined an average FPR value of 1.39 ± 1.45 FP/h across four patients.

A comparison between QD, BE, and HT indicates that the non-optimal policies BE and HT can achieve shorter delays, but the number of false positives rapidly increases, which may make these methods unsuitable for clinical application. While the average delay was 5.32 ± 11.78 s and 1.68 ± 7.55 s for BE and HT, respectively, the FPR was 3.0 ± 3.46 and 3.0 ±3.99 FP/h, respectively. Also, the performance of the HT was influenced by the noise in the sequence of *σ*_1_ values, resulting in 0% sensitivity in one patient (i.e., no seizure was correctly detected) and an average value of 82.5% across the whole population.

These results depend on the performance goals imposed by the cost (9). By increasing the penalty for detection delay in (9) (i.e., increasing *γ*), the QD was able to reduce the delay to the values achieved via BE (16 s, Fig. 6B, black dash-dot line), while keeping a lower number of FPs (7 vs. 11). By decreasing *γ*, instead, we achieved higher robustness to early modulations of *π*_*k*_, which can be due to abrupt spikes in the sequence of *σ*_1_, and were able to decrease the number of FPs and detect a seizure with less anticipation than the other methods (Figs. 7A, B).

**Fig. 8:**
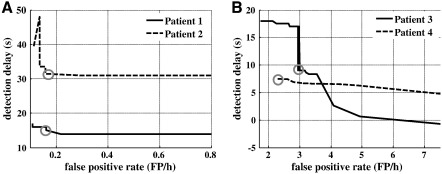
Sensitivity analysis. The average detection delay and false-positive rate for patients 1 and 2 (A), 3 and 4 (B) are computed for γ ranging from 0.01 to 0.999 on the validation data. Gray circles denote the average performance achieved with the value of γ used in Tables 2 and Table 3.

A sensitivity analysis of the QD policy to variations in the parameter *γ* is reported in Fig. 8. The policy was implemented for several values of *γ* in [0, 1], and for each value, the average delay and FPR per patient were estimated on the validation data. Results indicate that delays are quite insensitive to modulations in *γ* in patients 1 and 2, and low FPR values (< 0.17 FP/h) can be achieved while keeping the delay to the minimum value. However, in patient 4, delays were generally small (< 10 seconds) for FPRs below 2 FP/h, and further reductions in delay rapidly increase the FPR. Finally, performances for patient 3 was similar to that for BE and HT in terms of FPRs, presumably because of the less significant modulation of *σ*_1_ in the ictal versus nonictal state (Fig. 4).

## 4 Discussion

In this study, we propose a multichannel statistic that measures linear dependencies among all recorded sites of the epileptic brain simultaneously by combining power spectrum analysis and matrix theory. This statistic (the maximum singular value *σ*_1_ of the cross-power-based connectivity matrix) summarizes the change in topology that occurs in the brain network at the seizure onset and shows significantly different dynamics in ictal versus nonictal periods.

Using this statistic, we developed a computational framework for the AOSD. This framework combines Bayesian estimation (we use the a posteriori probability *π*_*k*_) with optimal control (we minimize the cost function $J_{0}$ in (10)) and provides a threshold-based detection policy, where the threshold varies with time based on the dynamics of *σ*_1_ and *π*_*k*_.

### 4.1 Multichannel versus single-channel statistics

Several statistics, both single channel and multichannel, have been computed from iEEG signals in the last 20 years to capture changes occurring in the brain network at seizure onset [6–33]. Although these statistics show some modulation between different nonictal and ictal states, they have a few drawbacks: (i) The statistics are usually computed on single channels [6–9,11,28,30–33] or a small subset of channels [10,29] from the focal area, which means that the foci must be known a priori with reasonable accuracy (i.e., localization and detection are correlated problems). This is less stringent with the multichannel statistics, where the only requirement is that the electrode grid is large enough to include the focal areas. (ii) The nonlinear multichannel statistics usually outperform linear single-channel and two-channel measures [60], but require larger amounts of data and computation. (iii) The modulation of these statistics around the seizure onset may vary with the subject or during the sleep/wake cycle [61], resulting in less predictable patterns.

These limitations can be (at least) in part addressed by increasing the number of combined channels and computing simple measures off of large enough matrices (on the order of hundreds of channels). Such a combination can provide more information about the brain network (albeit still incomplete) and exploits both spatial and temporal features, while the computation of the maximum singular value *σ*_1_ captures the overall degree of (linear) dependency among different brain sites and the sampled network topology. These dynamics were similar in all the patients in our data set, with clear differences during ictal and nonictal periods (Fig. 4) and a postictal resetting pattern that may last several minutes to a few hours.

These results positively affected the dynamics of the Bayesian a posteriori probability of an ictal state (*π*_*k*_). This probability evolves recursively with (7) and is used as a marker of the actual brain condition in the QD policy. Depending on the evolution of the maximum singular value, this probability clearly separates the ictal and nonictal periods, contributing to the success of the QD policy (Fig. 5). As indicated in Figs. 6 and 7, this a posteriori probability selectively increases at the seizure onset and remains high during the ictal period, before finally decreasing to zero. *π*_*k*_ rises to 1 (i.e., its maximum value) quickly at the seizure onset and usually has minor modulations outside the ictal period (Figs. 6 and 7). For this reason, the detection delay with QD is comparable to that achieved via BE, although the FPR is significantly lower in each patient with QD. The FPR is larger in BE because of the occurrence of abrupt spikes in the sequence of *π*_*k*_ that trigger false positives. These abrupt spikes in *π*_*k*_ may be caused by fluctuations or noise in the measure of the maximum singular value *σ*_1_. In this case, an adaptive threshold allows increasing the performance by selectively avoiding these peaks (see below).

### 4.2 Quickest detection policy

The proposed QD policy combines several well-known topics in abrupt change-point detection theory (e.g., quickest detection [35,36], bayesian estimation with loss function [58], dynamic programming [59], HMM [54]), generalizes the framework to the case of history-dependent sequential observations (which may be relevant for neural data and iEEG recordings [37], etc.), generalizes the framework to the case of history-dependent sequential observations (which may be relevant for neural data and iEEG recordings [37]), and results in an adaptive, unsupervised, threshold-based detection strategy that can potentially be implemented online.

Differently from current AOSD paradigms, our QD policy constructs the threshold by explicitly minimizing a cost function of the desired detection performance goals. Many current detectors track the modulation of a statistic computed from iEEG recordings over time, and then set a threshold on the statistic to estimate the ictal onset time [6,10–15]. The choice of the threshold is *supervised* and usually dependent on the fluctuations of the statistic, the specific patient, or the electrode position, and may require long training sessions to be more accurate. In our approach, instead, an *unsupervised* adaptive threshold falls out of the methodology and adapts according to the cost function, the model of the multichannel statistic (HMM), and current and past measurements.

More recent detection schemes that exploit classifiers (e.g., [16–33]), based on either ANN, SVM, or PCA, also generate unsupervised criteria that separate the feature space (statistic space) into dominant ictal and nonictal regions. Although optimization methods are used for separating ictal and nonictal data, the clustering procedure exploits the distance (in a specific high-dimensional geometric feature space) between the data, but does not encompass penalties for specific performance goals (e.g., minimize FPR).

In summary, the detection approaches proposed thus far follow a “bottom-up” flow, i.e., they determine criteria that most likely separate ictal and nonictal data, and then apply such criteria on sequential neural measurements and evaluate detection performance. Our QD policy, instead, follows a “top-down” approach, i.e., it requires a cost function that explicitly accounts for different performance goals (e.g., low probability of false positives, low distance between actual and detected seizure onset time, low probability of late detection, etc.) and then defines the detection strategy to minimize the cost function. Depending on the specific application (e.g., online detection of a clinical seizure, offline investigation of the electrographic onset of a known clinical seizure, etc.), the QD can be tuned to achieve a specific goal and reach the required level of sensitivity and specificity (Fig. 8).

Our QD policy also exploits a model-based control approach to seizure detection. Here, the QD formulation was derived for a two-state HMM representation of brain activity in ictal versus nonictal state. HMMs have been successfully used in several fields in statistics and engineering (for an overview, see [54]), and recently introduced for modeling neural data in sensitive tasks and neural prosthetics (e.g., [62,63]). In the case of seizure detection, the transition from a nonictal to an ictal state can be suitably treated as a hidden state transition, where the hidden state is the actual brain condition, which is unknown and partially sampled with electrodes. According to this interpretation, an epileptic brain can sequentially transition into *m* > 2 states, depending on the actual physiological conditions (e.g., the patient is awake or sleeping), the type of epilepsy, or the type of seizure occurring. The number of HMM states is generally patient specific and may vary with the available data. However, the framework outlined in (1)–(10) is general and can be extended to the case of HMMs with *m* > 2 states (e.g., imagine a separate *m*-state HMM for the nonictal periods and an *n*-state HMM for the ictal periods).

It is interesting that for AOSD, a minimal HMM with two states appears to be enough and led to low FPRs (< 0.2 FP/h) in two of the four patients in our data set. This may stem from the clear patterned dynamics of the maximum singular values in ictal versus nonictal periods, which is captured by the GLM structure in (5) and (6) (Fig. 5). GLM and maximum likelihood methods have been widely used before in the analysis and simulation of neuronal spike train analysis for several types of neural disorders [55,64,65] and provide a flexible framework for both stationary and nonstationary analyses. In our case, the GLM parameters are able to accurately capture changes that occur in the maximum singular value as soon as the seizure starts, and require a minimal set of training data to be estimated (only one seizure and 3 hours of nonictal data) in both conditions.

We are aware, however, that in the remaining two patients the FPR was quite high (> 2 FP/h). In this case, it is possible that the training data was not enough for modeling additional (slower) rhythms in the maximum singular value during the interictal state. Such additional dynamics presumably depend on the location of the focal region, which is not temporal in Patient 3-4, and the specific type of seizures. Indeed, while Patient 1 and 2 had distinct and large complex partial and tonic-clonic seizures, respectively, Patient 3-4 had short complex partial seizures with minor clinical evidence and simple partial seizures, respectively (Table 1). Another possible reason could be the spectral selectivity of our model, i.e., the fact that we consider only one frequency band per patient. Finally, a possible reason could be the limited number of states in our HMM, which could not be enough for these patients. These reasons led to a lack of model accuracy in patient 3 and 4, which presumably caused an increased fluctuation of the state variable *π*_*k*_ with frequent erroneous peaks and ultimately decreased the QD performance. Better results could be achieved by improving the HMM–GLM model framework with further information about the type of epilepsy and patient behavior.

Another aspect of the QD results in Tables 2 and 3 that needs to be addressed is the excessive detection delay (> 10 s) for a few seizures, which would not be useful for clinical application. Also, we note that the delay with QD was longer than with the HT and BE detector for several seizures, although the FPR was significantly lower.

The results of QD versus those of BE and HT are a consequence of the specific cost function that we defined, which penalizes the probability of false positives and very late detections more severely than detection delays in the order of 10–20 s. Our choice was motivated by the limited duration of the available recordings (less than 2 days per patient) and definitively resulted in a conservative policy, i.e.,the threshold *F*_*k*_(∙) ≫ 0, and, therefore, a seizure was detected only when the state variable *π*_*k*_ ≫ 0 (Figs. 6B, 7).

The issue of excessive delay may also be due to the spectral selectivity of the adopted connectivity. This can be a limitation as the seizures are characterized by several features (e.g., fast rhythmic activity, electrodecremental activity, isolated spikes, etc.), which may occur across multiple frequency bands. In this case, observations in a single frequency band would lead the multichannel statistic to late modulation and therefore a delayed detection.

A possible solution to this issue could be combining multichannel statistics across several frequency bands by combining maximum singular values of connectivity matrices computed in different frequency bands. Another solution to be explored is the use of nonlinear functions of the detection delay *T*_*QD*_− *T* in the cost function (9). In the current formulation, the penalty for delay in (9) grows quadratically with delay. We could allow this penalty to grow exponentially in delay (*e*^(*T*_*QD*_− *T*)^ ), and as long as the function is a non-decreasing function of the delay, the QD method will hold.

Future work entails validating our preliminary findings on larger data sets and reducing the detection delays so that they may be actionable for clinical intervention. We will also make direct comparisons with current AOSD approaches on the same set of statistics so that we may understand the degree of performance improvement we achieve with the QD methodology.

## Conflict of interest statement

The authors declare that there are no conflicts of interest.

## Figures and Tables

**Table 1:** Seizure origin, number of channels, duration of recordings, sampling frequency, and frequency band used for estimation of the connectivity matrix.

Patient	Sex	Seizure origin	Types of seizures	# iEEG channels	Hours of recordings	Sampling frequency (Hz)	Frequency band for σ_1_
1	Male	T	CP	34	40	500	13–30
2	Female	T	TC	28	47	500	4–7
3	Male	F	CP	44	47	250	13–30
4	Male	O	SP	26	34	500	13–30

*Note*. For each patient, the frequency band was chosen by maximizing the distance between ictal and nonictal generalized linear models (GLM) parameters (training data only).

**Table 2:**
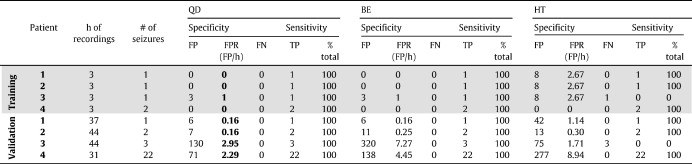
Performance analysis.

*Note*. FP, false positive; TP, true positive; FN, false negative; FPR, false positive rate.

**Table 3:**
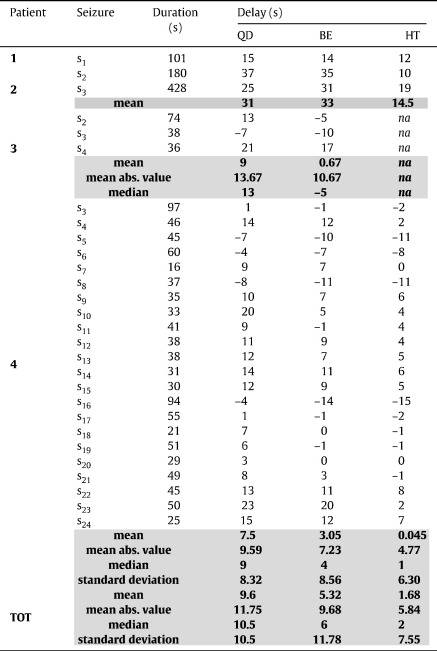
Detection delay on validation data.

*Note.* Positive and negative values denote delay and anticipation, respectively.

^a^Mean of the absolute values of delays and anticipations.

## Appendix Recursion of *π*_*k*_

Let us find the recursion equation for *π*_*k*_ as a function of *π*_*k*− 1_ in a *m* = 2 HMM. We start with stage 0:

$$\pi_{0}=\text{Pr}\left(x_{0},=,1,\text{|},z_{0}\right)=\text{Pr}\left(x_{0},=,1,\text{,},z_{0}\right)/\text{Pr}\left(z_{0}\right)=p_{1}q_{1}(z_{0})/\left(p_{1}q_{1}\left(z_{0}\right)+\left(1-p_{1}\right)q_{0}\left(z_{0}\right)\right)\text{.}$$

$$\pi_{1}=\text{Pr(}T\leq 1\;\text{|}z_{0},z_{1})=\text{Pr}\left(T\leq 1\text{,}\;z_{0},z_{1}\right)/\text{Pr}\left(z_{0},z_{1}\right)=\text{Pr}\left(T\leq 0\text{,}\;z_{0},z_{1}\;or\;T=1\text{,}\;z_{0},z_{1}\right)/\text{Pr}\left(z_{0},z_{1}\right)\;$$

$$=\text{Pr}\left(T\leq 0\text{,}\;z_{0},z_{1}\right)+\frac{\text{Pr}\left(T=1\text{,}\;z_{0},z_{1}\right)}{\text{Pr}\left(z_{0},z_{1}\right)}\;$$

$$=\frac{\text{Pr}(z_{0}\text{,}\;z_{1}|T\leq 0)\text{Pr}\left(T\leq 0)+\text{Pr}(z_{0}\text{,}\;z_{1}|T=1)\text{Pr}(T=1\right)}{\text{Pr}\left(z_{0},z_{1}\right)}$$

$$=\text{Pr}\left(z_{1}\text{|}T\leq 0,z_{0}\right)\text{Pr}\left(z_{0}\text{|}T\leq 0\right)/\text{Pr}\left(z_{1}|z_{0}\right)+\text{Pr}(z_{1}|T=1,z_{0})\text{Pr}(z_{0}|T=1)\text{Pr}(T=1)/\text{Pr}\left(z_{1}|z_{0}\right)$$

$$=\frac{\text{Pr}\left(T\leq 0\text{|}z_{0}\right)\text{Pr}\left(z_{1}\text{|}z_{0}\text{,}\;T\leq 0\right)+\text{Pr}\left(T=1\text{|}z_{0}\right)\text{Pr}\left(z_{1}\text{|}z_{0},T=1\right)\;}{\text{Pr}\left(z_{1}|z_{0}\right)\text{Pr}(z_{0})}$$

$$=\frac{\pi_{0}q_{1}\left(z_{1}|z_{0}\right)+\left(1-\pi_{0}\right)p_{01}q_{1}(z_{1}|z_{0})}{\pi_{0}q_{1}\left(z_{1}\text{|}z_{0}\right)+\left(1-\pi_{0}\right)p_{01}q_{1}\left(z_{1}|z_{0}\right)+\left(1-\pi_{0}\right)\left(1-p_{01}\right)q_{0}\left(z_{1}\text{|}z_{0}\right)}\text{.}$$

Define the likelihood ratio

$$L_{1}≜\frac{q_{1}\left(z_{1}\text{|}z_{0}\right)}{q_{0}\left(z_{1}|z_{0}\right)}\text{,}$$

then

$$\pi_{1}=\frac{\left[L_{1}{\left[\pi \right]}_{0}+\left(1-\pi_{0}\right)p_{01}\right]}{L_{1}[\pi_{0}+\left(1-\pi_{0}\right)p_{01}]+\left(1-\pi_{0}\right)\left(1-p_{01}\right)}$$

Similarly, it is straightforward to show that for

$$L_{k}≜\frac{q_{1}(z_{k}|H_{k})}{q_{0}\left(z_{k}|H_{k}\right)}\text{,}$$

where *H*_*k*_ = {*z*_0_, *z*_1_, …, *z*_*k*− 1_}, then

$$\pi_{k}=\frac{\left[L_{k}{\left[\pi \right]}_{k-1}+\left(1-\pi_{k-1}\right)p_{01}\right]}{L_{k}\left[\pi_{k-1}+\left(1-\pi_{k-1}\right)p_{01}\right]+\left(1-\pi_{k-1}\right)\left(1-p_{01}\right)}≜\text{Φ}\left(\pi_{k-1},z_{k},H_{k}\right)\text{.}$$
